# Effect of Preeclampsia on Ultrastructure of Thyroid Gland, Hepatic Type 1 Iodothyronine Deiodinase, and Thyroid Hormone Levels in Rats

**DOI:** 10.1155/2021/6681491

**Published:** 2021-04-20

**Authors:** Yunlu Liu, Zhuping Xu, Yanqin Li, Wenyan Jiang, Ming Lan, Xiaojuan Xie, Yang Wang

**Affiliations:** ^1^Institute of Laboratory Animal Sciences, Sichuan Academy of Medical Sciences & Sichuan Provincial People's Hospital, Chengdu 610212, China; ^2^Department of Pharmacy, Sichuan Academy of Medical Sciences & Sichuan Provincial People's Hospital, Chengdu 610072, China; ^3^Department of Internal Medicine, Shuangliu District Hospital of Traditional Chinese Medicine, Chengdu 610200, China; ^4^Department of Obstetrics and Gynaecology, Shuangliu Maternal and Child Health Care Hospital, Chengdu 610200, China; ^5^School of Rehabilitation and Health Preservation, Chengdu University of Traditional Chinese Medicine, Chengdu 610032, China; ^6^Department of Research and Training, Shuangliu Maternal and Child Health Care Hospital, Chengdu 610200, China

## Abstract

**Background:**

Although hypothyroidism during pregnancy may develop grave outcomes for both mothers and offspring, management of which is still a challenge due to the insufficient understanding of this disease. The close correlation between hypothyroidism and preeclampsia is well documented, suggesting that preeclampsia is a potential risk factor for the development of maternal hypothyroidism. However, the exact role of preeclampsia in gestational hypothyroidism is still obscure.

**Objective:**

In this study, we explored the possible mechanisms of the effect of preeclampsia on thyroid function of maternal rats.

**Methods:**

Thirty pregnant rats were randomly divided into normal pregnancy control (NOP), preeclampsia (PE), and preeclampsia supplemented with amlodipine besylate (PEAml). NG-Nitro-L-arginine-methyl ester was used to induce preeclamptic symptoms. On gestational day 21, rats were sacrificed, and then, the ultrastructure of the thyroid gland, type 1 iodothyronine deiodinase (Dio1) expression, and serum-free thyroxine (FT_4_), free triiodothyronine (FT_3_), and thyroid stimulation hormones (TSH) were assessed.

**Results:**

Compared to NOP rats, results of PE rats showed that thyroid follicular cells' ultrastructure was damaged; both hepatic Dio1 mRNA and protein levels were decreased. Interestingly, these changes were ameliorated in PEAml rats. Additionally, FT_4_, FT_3_, and TSH levels have no significant differences among groups.

**Conclusion:**

These findings indicated that preeclampsia could disrupt synthesis, secretion, and metabolism function of thyroid hormones by damaging thyroid follicular cells and interfering Dio1 expression.

## 1. Introduction

The stability of thyroid function is essential for normal growth, development, and metabolism, particularly in the gestation period and growing fetus [1]. Thyroid disorders, particularly hypothyroidism, are a common syndrome in women of childbearing age and feature frequently in pregnancy [2, 3]. It is well established that pregnant women with hypothyroidism will result in substantially greater risks of detrimental pregnancy and offspring outcomes such as miscarriage, stillbirth, neonatal respiratory distress syndrome, and fetal central nervous development defect [4–6]; consequently, serious burdens can be brought to the family and the society. However, evaluation and management of pregnant women with hypothyroidism are challenging due to the lack of sufficient cognition on the occurrence and development of disease [7].

Preeclampsia is one of the most common severe complications of pregnancy. Significantly increased frequency of hypothyroidism can be detected in preeclamptic women [8–11] and was positively correlated with the severity and medical history of preeclampsia [12–14]. Furthermore, preeclampsia may also predispose to the development of hypothyroidism in later years after delivery [15]. It also has been suggested that attention should be paid to the monitoring of thyroid function in pregnant women with preeclampsia because of the higher risk of hypothyroidism [16]. From these considerable clinical evidences, the obviously close association between hypothyroidism and preeclampsia is established. The exact mechanism of hypothyroidism in preeclamptic women, however, remains obscure.

Adequate thyroid function largely depends on the ability of thyroid hormone synthesis, secretion, and metabolism [17–19]. Thyroid follicular cells are the basic structure of the thyroid gland and essential for principal function of the thyroid gland: the site for synthesis and secretion of the thyroid hormones [20]. In thyroid follicular cells, endoplasmic reticulum, mitochondria, and microvilli are major organelles involved in thyroid hormone synthesis; lysosomes provide degradation pathways for thyroid hormone secretion [21]. Thyroxine (T_4_) and triiodothyronine (T_3_) are hormones synthesized and secreted from the thyroid gland; T_3_ is the bioactive form of the thyroid hormone [22]. About 80% of circulating T_3_ is derived from the metabolic transformation: the extrathyroidal peripheral conversion of T_4_ to T_3_ [23]. The peripheral conversion is mainly catalyzed by type 1 iodothyronine deiodinase (Dio1) [24], which is predominantly expressed in the liver [25].

With the background information above, we sought to identify how thyroid function is influenced by preeclampsia. Therefore, we evaluated the ultrastructure of the thyroid gland, the hepatic Dio1 expression, and circulating thyroid hormone levels in pregnant rats with preeclampsia induced by NG-nitro-L-arginine-methyl ester (L-NAME), to provide reliable experimental evidences for revealing the underlying mechanisms of hypothyroidism in preeclampsia; we further observed whether these changes were improved after administration of amlodipine besylate, a commonly used antihypertensive agent.

## 2. Materials and Methods

### 2.1. Equipment

In this study, the following major equipment was used: BP-6A tail-cuff system (Taimeng Technology, Chengdu, China), Sorvall ST8 centrifuge (Thermo Fisher Scientific, MA, USA), KHBST-360 microplate reader (Kehua Bio-engineering, Shanghai, China), CL-2000i microparticle chemiluminescence analyzer (Mindray, Shenzhen, China), EMUC6 Ultramicrotome (Leica, Wetzlar, Germany), H-600IV transmission electron microscope (Hitachi, Tokyo, Japan), KZ-II high-flux tissue grinder (Servicebio, Wuhan, China), SLAN®-96s qPCR system (Shanghai Hongshi Medical Technology Co., Ltd, Shanghai, China), MS-PB magnetic stirrer (Servicebio, Wuhan, China), DYY-6C electrophoresis apparatus (Liuyi, Beijing, China), and photographic film (Kodak, NY, USA).

### 2.2. Chemicals

In this study, the following major chemicals were used: L-NAME was obtained from Meilun Biotechnology Co., Ltd, Dalian, China, while amlodipine besylate was purchased from Pfizer, Dalian, China.

### 2.3. Animals

Sprague-Dawley rats (280-300 g) were included in this study, purchased from the Laboratory Animal Centre of Chongqing Medical University, Chongqing, China. Rats were housed in SPF barrier animal facility (temperature, 20-24°C; humidity, 50-60%; 12 h light/dark cycle) and had access to the standard food and water ad libitum. All the experimental procedures followed the *Guide for the Care and Use of Laboratory Animals* and were approved by the Welfare and Ethics of Experimental Animals Committee of Institute of Laboratory Animal Sciences, Sichuan Academy of Medical Sciences & Sichuan Provincial People's Hospital (No. Lunshen2019-001). And the SPF animal facility was accredited by the Sichuan Provincial Laboratory Animal Management Committee.

### 2.4. Experimental Protocol

Each female rat was mated overnight, and spermatozoa were found in the vaginal smear which was defined as the first day of pregnancy.

On day 13 of pregnancy, pregnant rats were divided randomly into three groups: pregnant rats that received daily subcutaneous injection of normal saline and gavage of 0.5% CMC-Na (normal pregnancy control group, NOP, *n* = 10), pregnant rats that received daily subcutaneous injection of 250 mg/kg L-NAME and gavage of 0.5% CMC-Na (preeclampsia group, PE, *n* = 10), and pregnant rats that received daily subcutaneous injection of 250 mg/kg L-NAME and gavage of 0.5 mg/kg amlodipine besylate (preeclampsia+amlodipine besylate group, PEAml, *n* = 10). L-NAME was dissolved in sterile normal saline. Amlodipine besylate was suspended in 0.5% CMC-Na, and the human equivalent dosage of amlodipine besylate for rats was determined based on clinical usage and body surface area conversion. Subcutaneous injection of L-NAME and gavage of 0.5% CMC-Na or amlodipine besylate were lasted from gestational day 13 to 20.

Preeclamptic symptoms were detected on gestational day 20. Systolic blood pressure was measured by the tail-cuff noninvasive method [26]. Urine protein was measured by a semiquantitative (negative, +, ++, +++, and ++++) urine dipstick test for morning urine samples [27, 28].

On day 21 of pregnancy, all rats were anesthetized using intraperitoneal injection of 50 mg/kg pentobarbital sodium, and blood samples were obtained through abdominal aortic puncture, and serum was separated for measurement of thyroid hormones. After anesthesia and euthanasia, tissue samples from the thyroid gland were resected and fixed in 3% glutaraldehyde for ultrastructural analyses. Tissue samples from the liver were dissected and stored at -80°C until measurement of Dio1 expression.

### 2.5. Observation of Ultrastructure of Thyroid Gland

After being fixed with 3% glutaraldehyde, the thyroid gland tissue was immersed in 1% osmium tetroxide for postfixation, dehydrated in graded acetone (30, 50, 70, 80, 90, 95, and 100%), successively infiltrated in a graded series of buffers that contain the dehydrating agent and epoxy resin osmotic solution at a ratio of 3 : 1, 1 : 1, and 1 : 3, for 0.5-1 h each permeation, and then embedded in Epon 812 resin, cut into semithin cross-sections with optical localization, and proceeded into ultrathin sections, doubly stained with uranium acetate and lead citrate. The ultrastructure changes were observed and photographed using a transmission electron microscope (TEM) [29]. Blinding of the independent observer was achieved by consecutive numbering of each sample.

### 2.6. Detection of Dio1 Expression

qPCR and western blot were used to assess the Dio1 mRNA expression and protein expression, respectively.

Dio1 mRNA expression was detected by qPCR as follows: (1) Total RNA was extracted from the rats' liver samples using TRIzol (Invitrogen Life Technology, CA, USA). The quantity and quality of RNA were verified by NanoDrop 2000 (Thermo Scientific, MA, USA). (2) 1 *μ*g of extracted RNA was used to synthesize cDNA using ReverTra Ace® qPCR RT Master Mix (TOYOBO, Osaka, Japan), and the reverse transcription reaction was carried out at 37°C for 15 min, 98°C for 5 min, and 4°C using a thermocycler. (3) Quantitative PCR reaction was performed using SYBR® Green Real-time PCR Master Mix (TOYOBO, Osaka, Japan), sense primer, antisense primer, template cDNA, and PCR grade water. The reaction conditions were predenaturing condition of 95°C for 2 min, 95°C reaction for 15 s, and 58°C annealing and extension for 30 s with a total of 40 cycles. The primers are showed in Table 1. (4) Glyceraldehyde-3-phosphate dehydrogenase (GAPDH) was used as an internal standard, and all results were normalized to GAPDH, and relative mRNA expression was calculated by the 2^-*ΔΔ*^Ct method [30].

Dio1 protein expression was detected by western blot analysis as follows: The rat liver tissue was washed twice with ice-cold PBS to remove the blood stain, and 20 mg of the tissue was lysed using RIPA lysis buffer (Servicebio, Wuhan, China) supplemented with 1 mM of PMSF (Servicebio, Wuhan, China) to isolate the total proteins. Protein concentration was determined using a BCA kit (Servicebio, Wuhan, China). According to the total protein concentration, protein samples (50 *μ*g) were mixed with the loading buffer and denatured at 100°C for 5 min and then were separated by SDS-PAGE (Servicebio, Wuhan, China) and transferred onto a PVDF membrane at 220 mA for 60 min. Membranes were blocked with 5% skimmed milk for 1 hour, then incubated with Dio1 antibody (1 : 500 dilutions, Proteintech, Wuhan, China) and internal loading control *β*-actin (1 : 5000 dilutions, Servicebio, Wuhan, China) at 4°C overnight. After washing with TBST for 3 times, incubation followed with secondary antibody HRP labelled goat anti-rabbit IgG (1 : 5000 dilutions, Multisciences, Hangzhou, China) at room temperature for 30 minutes. After chemiluminescence (Servicebio, Wuhan, China), the gel imaging films were scanned, and the Alpha Image Software (Alpha Innotech Co., CA, USA) was used to analyze the optical density of the target bands.

### 2.7. Measurement of Serum Thyroid Hormones

TSH was measured by the ELISA assay. Both free T_4_ (FT_4_) and free T_3_ (FT_3_) were measured by chemiluminescence assay technique.

For the measurement of TSH, the Rat TSH ELISA Kit (Elabscience, Wuhan, China) was applied, which adopts the Sandwich-ELISA principle and contains the micro-ELISA plate precoated with an antibody specific to rat TSH. The detailed procedure is as follows: (1) Add 100 *μ*L standard working solution or rat serum sample to each micro-ELISA plate well and then incubate for 90 min at 37°C. (2) Remove the liquid out of each well, immediately add 100 *μ*L working solution of biotinylated detection antibody specific for rat TSH to each well, mix gently, and then incubate for 1 hour at 37°C. (3) Aspirate the solution from each well, add 350 *μ*L wash buffer to each well, and soak for 1~2 min, then aspirate the solution from each well and pat it dry against clean absorbent paper. Repeat this step 3 times. (4) Add 100 *μ*L of HRP conjugate working solution to each well and incubate for 30 min at 37°C. (5) Aspirate the solution from each well and repeat the wash process for 5 times as conducted in step (3). (6) Add 90 *μ*L of the substrate reagent to each well, incubate for about 15 min at 37°C, and protect the plate from light. (7) Add 50 *μ*L of stop solution to each well and determine the optical density (OD) of each well at once with a microplate reader set to 450 nm. The OD value is proportional to the concentration of rat TSH. And the concentration of rat TSH in the serum samples was calculated by comparing the OD values of samples to the standard curve.

Serum FT_4_ and FT_3_ were measured as follows: Put the test tubes containing serum samples to the sample placement position of the full-automated Mindray CL-2000i system. A biotin-streptavidin competitive chemiluminescence immunoassay was adopted in the system for the measurement of FT_4_ and FT_3_, with the apparatus and reagents used in this assay coming from Mindray (Mindray, Shenzhen, China).

### 2.8. Statistical Analysis

Data were expressed as mean values ± standard deviations. Statistical analyses were performed with SPSS statistical software (version 18.0 for Windows, IBM, USA). Data of groups were compared using a one-way analysis of variance (ANOVA) test or Wilcoxon rank sum test. The significance level of *P* < 0.05 was considered to indicate statistical significance.

## 3. Results

### 3.1. Systolic Blood Pressure and Urine Protein in Each Group

Changes in systolic blood pressure and urine protein were measured on day 20 of pregnancy (Table 2).

PE rats were determined by an extremely significantly higher systolic blood pressure and urine protein compared to those in NOP rats (*P* < 0.01), while systolic blood pressure and urine protein from PEAml rats were significantly ameliorated compared with PE (*P* < 0.01) and returned towards normal levels.

### 3.2. Effect of Preeclampsia on Ultrastructure of Thyroid Gland

**Figure 1:**
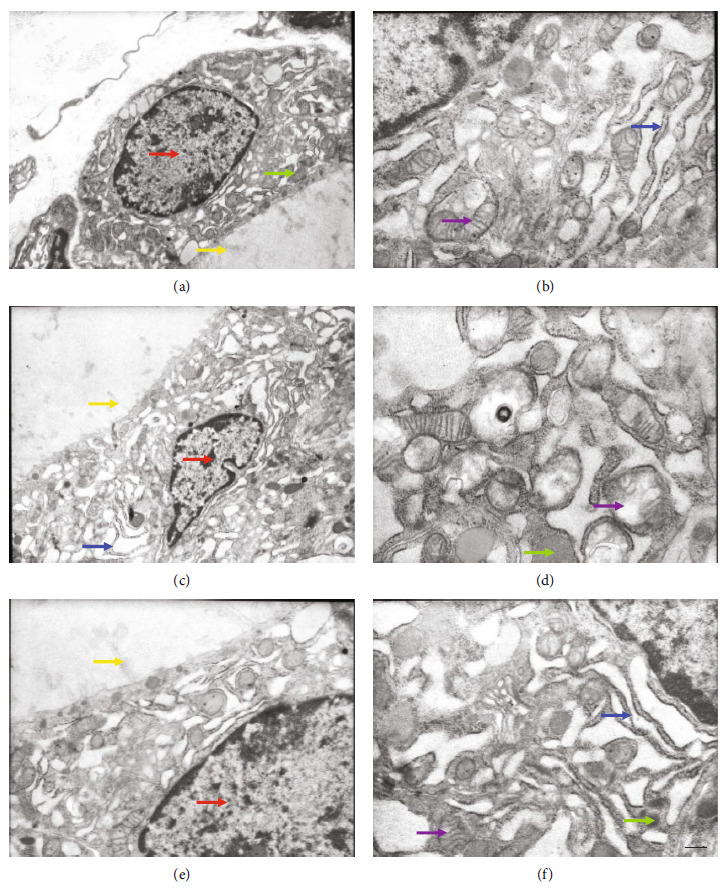
Effect of preeclampsia on the ultrastructure of thyroid follicular cells in rats. Measured by TEM analysis. (a, b) Represent NOP group; (c, d) represent the PE group; (e, f) represent the PEAml group, scale bar = 1 *μ*m. Red arrow: nucleus; blue arrow: rough endoplasmic reticulum; purple arrow: mitochondria; green arrow: lysosome; yellow arrow: microvilli. *n* = 4-6.

The thyroid follicular cells of NOP were observed under an electron microscope. The nucleus was oval, and the chromatin was evenly distributed in the nucleus. Abundant rough endoplasmic reticulum, mitochondrion, ribosome, colloidal granule, lysosome, and so on can be seen in the cytoplasm with a clear structure and tight junctions. A few microvilli were neatly arranged on the cell surface (Figures 1(a) and 1(b)).

The thyroid follicular cells of PE were observed under an electron microscope. The nucleus was irregular, and the chromatin was aggregated in the nucleus. Many markedly dilated rough endoplasmic reticula and some swollen, cristae broken mitochondria were showed in the cytoplasm. Lysosome decreased. Ribosome, colloidal granules, and so on were also noticed. Large vacuoles and short, blunt, sparse microvilli were depicted in some cells (Figures 1(c) and 1(d)).

The thyroid follicular cells of PEAml were observed under an electron microscope. The nucleus was oval, and the chromatin in the nucleus was mildly aggregated. Slightly expanded rough endoplasmic reticulum and slightly swollen mitochondria were observed in the cytoplasm. Lysosomes and ribosomes with intact structures were also revealed in the cytoplasm. A few microvilli with well-developed borders were exhibited on the cell surface (Figures 1(e) and 1(f)).

### 3.3. Effect of Preeclampsia on Dio1 Expression in the Liver

**Figure 2:**
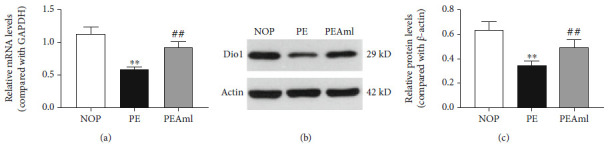
Effect of preeclampsia on mRNA and protein levels of hepatic Dio1 in rats. Measured by qPCR and western blot, respectively. (a) The mRNA expression of hepatic Dio1 in the NOP, PE, and PEAml groups. (b) The typical protein bands of hepatic Dio1 in the NOP, PE, and PEAml groups. (c) The protein expression of hepatic Dio1 in the NOP, PE, and PEAml groups. ^∗∗^vs. NOP, *P* < 0.01; ^#^vs. PE, *P* < 0.05; ^##^vs. PE, *P* < 0.01. *n* = 10 per group.

Changes in hepatic Dio1 mRNA expression were analyzed (Figure 2(a)). The obtained results indicated that Dio1 mRNA expression levels were significantly decreased by 49.1% in PE rats than that in NOP rats (*P* < 0.01), while PEAml rats did increase the Dio1 mRNA expression level by 62.1% relative to that of PE animals (*P* < 0.01).

Changes in hepatic Dio1 protein expression were detected (Figures 2(b) and 2(c)). Consistent with the decrease of Dio1 mRNA expression, the expression of the encoding protein Dio1 in rats' livers was significantly downregulated after L-NAME exposure (PE) compared with NOP rats (*P* < 0.01): grey value analysis showed that in NOP, the protein Dio1 was 0.65 ± 0.19-fold of reference protein ACTIN, while in PE, the ratio of Dio1/ACTIN was 0.34 ± 0.13, indicating that Dio1 protein expression reduced by 47.7%. Compared with the PE, Dio1 in PEAml rats were significantly elevated by 32.1% (*P* < 0.05), which was 0.5 ± 0.19-fold of reference protein ACTIN.

### 3.4. Effect of Preeclampsia on Serum Levels of Thyroid Hormones

Changes in TSH, FT_4_, and FT_3_ were measured, and no significant difference between groups was observed (*P* > 0.05) (Table 3).

## 4. Discussion

Many previous clinical studies have demonstrated that preeclampsia can result in thyroid dysfunction in pregnant women and had a significant adverse impact on maternal and fetal outcomes [31]. However, to the best of our knowledge, the specific mechanism of the effect of preeclampsia on hypothyroidism still needs to be elucidated. In this study, the significantly increased systolic blood pressure and urine protein in PE group rats induced by L-NAME are in accordance with the preeclamptic symptoms in the clinic [28]. According to the successful established animal model of preeclampsia, we investigate the underlying mechanism of the effect of preeclampsia on thyroid function.

Thyroid follicular cells are important functional units of the thyroid gland and the place of thyroid hormone synthesis and secretion; the stability of its structure directly affects the ability of thyroid hormone synthesis and secretion [32, 33]. We, therefore, investigated the ultrastructure changes in thyroid follicular cells under the preeclamptic status. By using a transmission electron microscope, findings showed that preeclampsia may damage the nuclei and various organelles. As the preeclamptic syndrome occurred, the nucleus of follicular cells started to be irregular and dilation of rough endoplasmic reticulum appeared; therefore, the synthesis of thyroglobulin (Tg) is reduced. And it is acknowledged that thyroid hormones were formed by iodinating and coupling the tyrosyl residues of Tg mediated by thyroid peroxidase [21]. Moreover, swollen mitochondria can reduce ATP production by seriously affecting the ability of mitochondrial respiration and oxidative phosphorylation [34]. Microvilli are not only the place where oxidization of iodide occurs but also the main structure to assist the iodized colloidal particles in the follicular lumen to reabsorb into the follicular epithelium and form the colloid vesicle (i.e., Tg); the abnormal microvilli meant the decreased function of iodination and transportation and further affect the synthesis of thyroid hormones [35, 36]. On the other side, the decreased lysosomes in the follicular epithelium can adversely affect degradation ability of which to the colloid vesicle, which leads to the fewer amount of T_3_ and T_4_ secreted into blood [37]. We can clearly see that these microscopic changes could result in deleterious effect on the thyroid hormone synthesis and secretion ability, and interestingly, all these ultrastructural damages were relieved after the antihypertensive treatment of preeclampsia.

T_3_ is the bioactive form of thyroid hormone, which exerts powerful and rapid intracellular action. The metabolism of extrathyroidal T_3_ is critical to the homeostasis of thyroid hormones in the body's environment [38]. Approximately 80% peripheral T_3_ are derived from T_4_ outer ring deiodination [39]. Hepatic Dio1 is a major enzyme with outer ring deiodinase activities, which is essential in the tissue-specific regulation of thyroid hormone bioactivity [3]. According to this, to fully investigate how preeclampsia disturbed thyroid function at the metabolic level, we detected the hepatic Dio1. Results showed that both mRNA expression and protein expression of Dio1 were significantly decreased under preeclamptic conditions in rats' livers and remarkably upregulated after treatment with the antihypertensive drug, suggesting that the downregulated Dio1 expression was caused by preeclampsia; as a result, the metabolism of thyroid hormones can be affected which may lead to decreased hepatic T_3_ production.

Most of the T_4_ and T_3_ are carried by thyroxine-binding globulin (TBG) in blood, resulting in less than 0.05% of them remaining free [33]. FT_4_ and FT_3_ are hormones that enter target cells and bind to receptors to elicit biological effect. Moreover, in pregnant status, the TBG is stimulated by human chorionic gonadotropin (HCG), which can affect the levels of total T_4_ and total T_3_; however, serum FT_4_ and FT_3_ are less affected [33, 40]. In addition, TSH is a hormone that can promote the release and synthesis of T_4_ and T_3_, also a key role in the thyroid function negative-feedback loop [41]. Thus, it was necessary for us to measure serum FT_4_, FT_3_, and TSH, which were valuable indicators with high sensitivity for reflecting overall thyroid function [42]. It was observed that serum TSH, FT_4_, and FT_3_ levels had no significant changes in preeclamptic rats. We speculated that the reason why these changes were not statistically significant may involve TBG, a combined protein produced in the liver and having high affinity with thyroid hormones [43]: firstly, the gestational period of rats is short, only about 21 days [44], and the modelling was started on day 13 of pregnancy. It was reported that TBG had a half-life of more than 5 days [45]. Therefore, the remaining uncleared circulating TBG still continues to produce free thyroid hormones in the late stage of modelling. In addition, preeclampsia could deleteriously affect the liver [46]; consequently, both the liver TBG production and binding ability of TBG might be interfered [47], therefore raising the fraction of free thyroid hormones in serum, thus, overall, counteracting the decrease of the thyroid hormone caused by the impairment of thyroid synthesis, secretion, and metabolic transformation ability in the preeclamptic model. Unfortunately, we did not evaluate TBG to confirm this hypothesis.

To objectively assess the effect of preeclampsia on thyroid function, we especially designed the PEAml group, and amlodipine besylate (Aml), as an antihypertensive drug, was used to treat preeclampsia in this group. Results showed that compared with the PE group, the preeclamptic symptoms, the thyroid ultrastructure damage, and the decreased Dio1 expression were ameliorated in the PEAml group. Aml has already demonstrated that the therapeutic efficacy of which was exerted through potentiating vascular endothelial growth factor (VEGF) signaling pathways [48–50]. The main function of VEGF is to regulate vascular tone, trigger angiogenesis, accelerate proliferation, and repair epithelial cells [51], consequently playing an important role in regulating and maintaining vasculature in normal organs [52]. Previous studies have demonstrated that the occurrence of preeclampsia was tightly linked with the decreased bioavailability of VEGF [53–55]. Moreover, results of our study and others [56, 57] have also showed that the preeclamptic symptom could be relieved after the treatment of Aml. Additionally, in the vascular endothelium of the thyroid gland and liver, VEGF has been found to be highly expressed; the suppression of VEGF cellular signaling pathways can result in vascular disturbances and even regression in these organs [58–60]. We therefore speculate that preeclampsia could develop blood vessel injury of the thyroid gland and liver via disturbing VEGF signaling pathways, leading to impairment of the thyroid ultrastructure and expression of hepatic Dio1, further interfering the synthesis, secretion, and metabolism of thyroid hormones; the results of the PEAml group prove this speculation, which reminds us that special attention should be paid to the protection of the thyroid gland and liver in preeclamptic women, to avoid the onset of hypothyroidism. However, more detailed analysis should be involved to identify the exact role of VEGF during the pathologic process of hypothyroidism caused by preeclampsia.

## 5. Conclusions

Taken together, by investigating the multiple aspects of thyroid functional status in L-NAME-induced preeclamptic rats, our results demonstrated for the first time that the ultrastructure of thyroid follicular cell and hepatic Dio1 expression could be significantly impaired by preeclampsia, and all the damages were ameliorated after antihypertensive therapy, which provides the reliable evidence for understanding the underlying mechanism of hypothyroidism in preeclamptic condition.

## Figures and Tables

**Table 1:** The prime information of GAPDH and Dio1.

Genes	Primer	Sequences (5′-3′)	Product size (bp)
GAPDH	R-GAPDH-S	GGTGCTGAGTATGTCGTGGAGT	105
	R-GAPDH-A	GGAAGGGGCGGAGATGA	

Dio1	R-Dio1-S	GGTGGACACAATGCAGAACCA	114
	R-Dio1-A	TAGTTCCAAGGGCCAGGTTT	

**Table 2:** Systolic blood pressure (SBP) and urine protein.

Parameters	NOP	PE	PEAml
SBP (mm Hg)	89 ± 11.6	143 ± 14.8^∗∗^	97 ± 6.3^##^
Urine protein (grades)	Negative	+ to ++++^∗∗^	Negative to +^##^

Data are mean values and standard deviations. ^∗∗^ vs. NOP, *P* < 0.01; ^##^vs. PE, *P* < 0.01. *n* = 10 per group.

**Table 3:** Serum levels of TSH, FT_4_, and FT_3_.

Parameters	NOP	PE	PEAml
TSH (ng/mL)	5.5 ± 1.1	5.3 ± 1.5	5.0 ± 2.1
FT_4_ (pmol/L)	29.2 ± 2.0	29.3 ± 2.6	29.9 ± 2.1
FT_3_ (pmol/L)	2.8 ± 1.9	2.8 ± 1.3	3.3 ± 1.2

Data are mean values and standard deviations. *n* = 10 per group.

## Data Availability

The initial data used to support the findings of this study are available from the corresponding author upon request.

## References

[1] Liu Z., Chen Y., Chen G. (2019). Impaired glucose metabolism in young offspring of female rats with hypothyroidism. *Journal Diabetes Research*.

[2] Okosieme O. E., Marx H., Lazarus J. H. (2008). Medical management of thyroid dysfunction in pregnancy and the postpartum. *Expert Opinion on Pharmacotherapy*.

[3] Mullur R., Liu Y. Y., Brent G. A. (2014). Thyroid hormone regulation of metabolism. *Physiological Reviews*.

[4] Lazarus J. H., Kokandi A. (2000). Thyroid disease in relation to pregnancy: a decade of change. *Clinical Endocrinology*.

[5] Glinoer D. (2003). Management of hypo- and hyperthyroidism during pregnancy. *Growth Hormone & IGF Research*.

[6] Haddow J. E., Palomaki G. E., Allan W. C. (1999). Maternal thyroid deficiency during pregnancy and subsequent neuropsychological development of the child. *New England Journal of Medicine*.

[7] Glinoer D., Abalovich M. (2007). Unresolved questions in managing hypothyroidism during pregnancy. *BMJ*.

[8] Roncaglia N., Avagliano L., Crippa I. (2006). Preeclampsia is strongly associated with maternal hypothyroidism. *American Journal of Obstetrics and Gynecology*.

[9] Kumar A., Ghosh B. K., Murthy N. S. (2005). Maternal thyroid hormonal status in preeclampsia. *Indian Journal of Medical Sciences*.

[10] Harshvardhan L., Dariya S. S., Sharma A., Verma L. (2017). Study of association of thyroid hormone in pre-eclampsia and normal pregnancy. *The Journal of the Association of Physicians of India*.

[11] Alavi A., Adabi K., Nekuie S. (2012). Thyroid dysfunction and autoantibodies association with hypertensive disorders during pregnancy. *Journal of Pregnancy*.

[12] Basbug M., Aygen E., Tayyar M., Tutus A., Kaya E., Oktem O. (1999). Correlation between maternal thyroid function tests and endothelin in preeclampsia-eclampsia. *Obstetrics and Gynecology*.

[13] Williams D. (2011). Long-term complications of preeclampsia. *Seminars in Nephrology*.

[14] Mannisto T., Karumanchi S. A., Pouta A. (2013). Preeclampsia, gestational hypertension and subsequent hypothyroidism. *Pregnancy Hypertens*.

[15] Levine R. J., Vatten L. J., Horowitz G. L. (2009). Pre-eclampsia, soluble fms-like tyrosine kinase 1, and the risk of reduced thyroid function: nested case-control and population-based study. *Bmj-British Medical Journal*.

[16] Procopciuc L. M., Hazi G. M., Caracostea G. (2011). Correlation between the TSHRc-Asp727Glu polymorphism and plasma thyroid stimulating hormone levels in Romanian preeclamptic women. *Gynecological Endocrinology*.

[17] Larsen P. R. (1982). Thyroid-pituitary interaction: feedback regulation of thyrotropin secretion by thyroid hormones. *The New England Journal of Medicine*.

[18] Larsen P. R., Berry M. J. (1995). Nutritional and hormonal regulation of thyroid hormone deiodinases. *Annual Review of Nutrition*.

[19] Zaccarelli-Marino M. A., Alessi R., Balderi T. Z., Martins M. A. G. (2019). Association between the occurrence of primary hypothyroidism and the exposure of the population near to industrial pollutants in São Paulo State, Brazil. *International journal of environmental research and public health*.

[20] Eskalli Z., Achouri Y., Hahn S. (2016). Overexpression of interleukin-4 in the thyroid of transgenic mice upregulates the expression of Duox1 and the anion transporter pendrin. *Thyroid*.

[21] de Vijlder J. J. (2003). Primary congenital hypothyroidism: defects in iodine pathways. *European Journal of Endocrinology*.

[22] Ito M., Kawasaki M., Danno H. (2019). Serum thyroid hormone balance in levothyroxine monotherapy-treated patients with atrophic thyroid after radioiodine treatment for Graves' disease. *Thyroid*.

[23] Pilo A., Iervasi G., Vitek F., Ferdeghini M., Cazzuola F., Bianchi R. (1990). Thyroidal and peripheral production of 3,5,3'-triiodothyronine in humans by multicompartmental analysis. *The American Journal of Physiology*.

[24] Germain D. L. S., Galton V. A., Hernandez A. (2009). Minireview: defining the roles of the iodothyronine deiodinases: current concepts and challenges. *Endocrinology*.

[25] Lisboa P. C., de Oliveira E., Manhaes A. C. (2015). Effects of maternal nicotine exposure on thyroid hormone metabolism and function in adult rat progeny. *The Journal of Endocrinology*.

[26] Yallampalli C., Garfield R. E. (1993). Inhibition of nitric oxide synthesis in rats during pregnancy produces signs similar to those of preeclampsia. *American Journal of Obstetrics and Gynecology*.

[27] Shukla J., Walsh S. W. (2015). Neutrophil release of myeloperoxidase in systemic vasculature of obese women may put them at risk for preeclampsia. *Reproductive Sciences*.

[28] Ebose E. J., Campbell P. I., Okorodudu A. O. (2007). Electrolytes and pH changes in pre-eclamptic rats. *Clinica Chimica Acta*.

[29] Gao J., Lin X. Y., Liu X. H. (2013). Effect of combined excess iodine and low-protein diet on thyroid hormones and ultrastructure in Wistar rats. *Biological Trace Element Research*.

[30] Pfaffl M. W. (2001). A new mathematical model for relative quantification in real-time RT-PCR. *Nucleic Acids Research*.

[31] Zhou Q., Zhang Y., Zhou J. (2018). Analysis of detection results of thyroid function-related indexes in pregnant women and establishment of the reference interval. *Experimental and Therapeutic Medicine*.

[32] Darras V. M., Houbrechts A. M., Van Herck S. L. J. (2015). Intracellular thyroid hormone metabolism as a local regulator of nuclear thyroid hormone receptor-mediated impact on vertebrate development. *Biochimica Et Biophysica Acta-Gene Regulatory Mechanisms*.

[33] Xu Y., Wu D., Wu W. (2019). Diagnostic value of cytology, thyroglobulin, and combination of them in fine-needle aspiration of metastatic lymph nodes in patients with differentiated thyroid cancer: a systematic review and network meta-analysis. *Medicine (Baltimore)*.

[34] Werneck F. Z., Coelho E. F., de Lima J. R. (2014). Pulmonary oxygen uptake kinetics during exercise in subclinical hypothyroidism. *Thyroid*.

[35] Guo C., Chen X., Song H. (2014). Intrinsic expression of a multiexon type 3 deiodinase gene controls zebrafish embryo size. *Endocrinology*.

[36] Plantinga T. S., Tesselaar M. H., Morreau H. (2016). Autophagy activity is associated with membranous sodium iodide symporter expression and clinical response to radioiodine therapy in non-medullary thyroid cancer. *Autophagy*.

[37] Robbins J. (1976). Thyroxine-binding proteins. *Progress in Clinical and Biological Research*.

[38] Visser T. J. (1996). Pathways of thyroid hormone metabolism. *Acta Medica Austriaca*.

[39] Larsen P. R., Zavacki A. M. (2012). Role of the iodothyronine deiodinases in the physiology and pathophysiology of thyroid hormone action. *European thyroid journal*.

[40] Taylor P. N., Lazarus J. H. (2019). Hypothyroidism in pregnancy. *Endocrinology and Metabolism Clinics*.

[41] Aoun E. G., Lee M. R., Haass-Koffler C. L. (2015). Relationship between the thyroid axis and alcohol craving. *Alcohol and Alcoholism*.

[42] Saravanan G., Ponmurugan P. (2012). Antidiabetic effect of S-allylcysteine: effect on thyroid hormone and circulatory antioxidant system in experimental diabetic rats. *Journal of Diabetes and its Complications*.

[43] Soldin O. P., Goughenour B. E., Gilbert S. Z., Landy H. J., Soldin S. J. (2009). Thyroid hormone levels associated with active and passive cigarette smoking. *Thyroid*.

[44] Signore C., Ueland P. M., Troendle J., Mills J. L. (2008). Choline concentrations in human maternal and cord blood and intelligence at 5 y of age. *The American Journal of Clinical Nutrition*.

[45] YOUNG R. A., RAJATANAVIN R., MORING A. F., BRAVERMAN L. E. (1985). Fasting induces the generation of serum thyronine-binding globulin in Zucker rats. *Endocrinology*.

[46] Armaly Z., Jadaon J. E., Jabbour A., Abassi Z. A. (2018). Preeclampsia: novel mechanisms and potential therapeutic approaches. *Frontiers in Physiology*.

[47] Borzio M., Caldara R., Borzio F., Piepoli V., Rampini P., Ferrari C. (1983). Thyroid function tests in chronic liver disease: evidence for multiple abnormalities despite clinical euthyroidism. *Gut*.

[48] Champagne S., Hittinger L., Heloire F. (2002). Reduced coronary vasodilator responses to amlodipine in pacing-induced heart failure in conscious dogs: role of nitric oxide. *British Journal of Pharmacology*.

[49] Ronco M. T., Frances D., de Lujan Alvarez M. (2007). Vascular endothelial growth factor and nitric oxide in rat liver regeneration. *Life Sciences*.

[50] Sharma A., Trane A., Yu C., Jasmin J.-F., Bernatchez P. (2011). Amlodipine increases endothelial nitric oxide release by modulating binding of native eNOS protein complex to caveolin-1. *European Journal of Pharmacology*.

[51] Block H. S., Biller J., Biller J., Ferro J. M. (2014). Chapter 105 - neurology of pregnancy. *Handbook of Clinical Neurology*.

[52] Kamba T., McDonald D. M. (2007). Mechanisms of adverse effects of anti-VEGF therapy for cancer. *British Journal of Cancer*.

[53] Tsatsaris V., Goffin F., Munaut C. (2003). Overexpression of the soluble vascular endothelial growth factor receptor in preeclamptic patients: pathophysiological consequences. *Journal of Clinical Endocrinology & Metabolism*.

[54] Luttun A., Carmeliet P. (2003). Soluble VEGF receptor Flt1: the elusive preeclampsia factor discovered?. *The Journal of Clinical Investigation*.

[55] Ahmed A., Cudmore M. J. (2009). Can the biology of VEGF and haem oxygenases help solve pre-eclampsia?. *Biochemical Society Transactions*.

[56] deSwiet M. (1985). Antihypertensive drugs in pregnancy. *British medical journal (Clinical research ed.)*.

[57] Mito A., Murashima A., Wada Y. (2019). Safety of amlodipine in early pregnancy. *Journal of the American Heart Association*.

[58] Yamamoto C., Yagi S., Hori T. (2010). Significance of portal venous VEGF during liver regeneration after hepatectomy. *The Journal of Surgical Research*.

[59] Badizadegan K., Wolf J. L., Odze R. D., Goldblum J. R. (2009). CHAPTER 45 - liver pathology in pregnancy. *Surgical Pathology of the GI Tract, Liver, Biliary Tract, and Pancreas*.

[60] Kamba T., Tam B. Y. Y., Hashizume H. (2006). VEGF-dependent plasticity of fenestrated capillaries in the normal adult microvasculature. *American Journal of Physiology-Heart and Circulatory Physiology*.

